# Research on a new management model of distribution Internet of Things

**DOI:** 10.1038/s41598-024-51570-1

**Published:** 2024-01-10

**Authors:** Chao Chen, Liwu Gong, Xin Luo, Fuwang Wang

**Affiliations:** 1 State Grid Zhejiang Electric Power Co., Ltd., Jiaxing Power Supply Company, Jiaxin, 314000 Zhejiang Province China; 2 School of Electrical Engineering, Northeast Electric Power University, Jilin, 132000 Jilin Province China

**Keywords:** Information technology, Computer science

## Abstract

Based on the characteristics of controllable intelligence of the Internet of Things (IoT) and the requirements of the new distribution Network for function and transmission delay, this study proposes a method of combining edge collaborative computing and distribution Network station area, and builds a distribution Network management structure model by combining the Packet Transport Network (PTN) Network structure. The multi-terminal node distribution model of distributed IoT is established. Finally, a distribution IoT management model is constructed based on the edge multi-node cooperative reasoning algorithm and collaborative computing architecture model. The purpose of this paper is to solve the problem of large reasoning delay caused by heavy computing tasks in distribution cloud servers. The final results show that the model reduces the inference delay of cloud computing when a large number of smart device terminals of distribution IoT are connected to the network.

## Introduction

The distribution system itself has the characteristics of wide geographical distribution, various types of equipment, diverse network connections and changeable operation modes. With the application of distribution automation monitoring system, the heterogeneous and multivariate data generated by it increases exponentially, and the amount of data has reached the level of big data. The cloud server of Internet of Things (IoT) has a heavy computing task, so there are some problems, such as excessive reasoning delay and high redundancy of data acquisition, which affect the working efficiency of the system. Therefore, how to effectively collect the data of distribution Internet of Things, optimize the management structure of the distribution network and improve the efficiency of data processing has become an urgent problem to be solved in the construction of the distribution Internet of Things.

The information and communication technology of the IoT maintains the characteristics of ubiquitous sensing and Interworking Protocol (IP) communication, but also has decentralized sensing ability. It distributes intelligent processing units at different levels of the distribution network, and realizes comprehensive sensing and supervision of the distribution network and asset information through the combination of cloud computing and edge computing. The edge computing is used to reduce the problems of edge IoT device management, data acquisition and processing, and the large and complex data communication volume in cloud processing^1^. The application of edge computing in smart grid has attracted extensive attention and research from scholars at home and abroad, mainly focusing on energy monitoring and management, load forecasting and scheduling, security and privacy protection, multi-energy collaborative management, fault detection and maintenance and so on. Luo et al. proposed a short-term energy consumption prediction system based on edge computing architecture. The experimental results show that the system can provide high precision real-time energy consumption prediction support^2^. Liu et al. designed an IoT energy management system based on a deep reinforcement learning edge computing infrastructure. Simulation results show that compared with traditional systems, the proposed system can achieve lower energy consumption while reducing delay^3^. Xu et al. used the hash SHA256 algorithm to generate a set of code word groups as the index of records. When searching, users input multiple keywords and generate corresponding trap doors. The trap doors are accurately matched with the index, and all matching results are fed back to users. It can effectively protect the data stored at the edge of the power grid^4^. Bai et al. proposed a distributed distribution fault detection model based on edge intelligence, which introduced edge computing into the traditional cloud-based system, effectively reducing the fault analysis and processing time, and ensuring the safe and stable operation of the power communication network^5^. Cai et al. proposed an intelligent decision-making method for quick power supply restoration of distribution network based on cloud-edge collaboration, and proposed a new implementation scheme for quick power supply restoration of distribution networks^6^. Li et al. adopted edge computing technology to transfer computing and storage tasks from cloud computing centers to edge devices closer to data sources for processing, reducing data transmission delay and improving data processing speed and real-time. A special data processing framework for distribution network load multi-source, strong coupling data acquisition, cleaning and aggregation is constructed, and the energy efficiency management system of the distribution network is further constructed by studying the quality optimization of power grid and distributed energy scheduling^7^. Literature^8^ proposes a unified energy management architecture applied to distributed edge computing of renewable energy, which improves the response speed of distributed energy control. Literature^9^ constructed the edge computing architecture of smart grid model. Taking intelligent measurement data acquisition as an example, the role of edge computing in data analysis, its efficiency and security were analyzed in detail. Literature^10^ designed and realized the service collaboration of distribution IoT based on the pipeline mode computing model around the edge computing requirements, which could reduce the computational load, but the computational speed was not studied. Literature^11^ takes intelligent terminals of power distribution as the core, provides collaborative data assistance based on edge computing, and realizes quantitative calculation and intelligent identification of local topology data in low-voltage station areas. In the literature^12^, the whole distribution station area is taken as the edge computer node, the characteristic indicators of the station area are established through monitoring data, and the weight is selected to judge the operation state of the station area, so as to further reduce the computational redundancy. In literature^13^, the collaborative management architecture of the power company's jurisdiction area is designed based on edge computing technology for the IoT in the distribution area. In literature^14^, large-scale distributed co-simulation technology is applied to smart grid system and a new co-simulator is designed. Literature^15^ and literature^16^ used edge computing technology to design diversified load management, optimized the data processing flow, and proposed solutions to the heterogeneous problem of intelligent terminal devices in the distribution IoT. The above researches show that edge computing can be effectively applied to smart distribution networks, but most of the research focuses on the design of distribution network management structure, and the optimization operation and efficient work of distribution Internet of Things are not studied in depth.

Edge computing extends from cloud computing to intelligent terminals and edge nodes of the Internet of Things, which can improve computing speed and processing efficiency. A large number of sensing and computing terminals are arranged in the station area. By using the data acquisition and processing capabilities of these edge devices and the interactive capabilities with users, the information is preprocessed, which saves computing resources for the cloud computing center. The two can be unified into a whole, and the precise control and real-time response of the entire distribution IoT can be realized^17,18^. According to the dimensions of collaborative computing between cloud computing and edge computing, cloud edge collaboration can be divided into the following four categories: (1) resource collaboration; (2) data collaboration; (3) intelligent collaboration; (4) service collaboration. Based on the above four categories^19^. This paper studies the model of reducing inference delay in the process of distributed IoT cloud computing, designs a multi-terminal distribution model of distribution IoT, and constructs a distribution IoT management model based on edge multi-node collaborative computing, thus completing the inference optimization task of distributed cloud computing.

## Edge computing and distributed Internet of Things

### Edge computing

**Figure 1:**
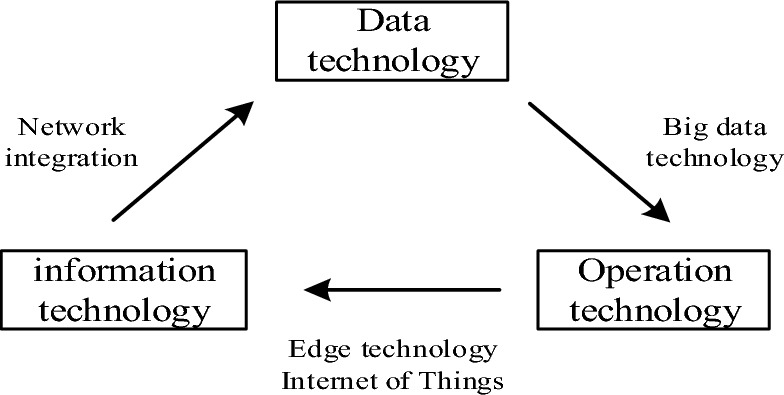
Schematic diagram of edge computing positioning.

Through the integration of computing, communication and control technologies, the intelligent terminal of IoT has formed an organic whole of information system and physical systems^20^. There are some shortcomings in the current calculation methods of distribution networks, such as fast calculation, data management, data terminal integration, analysis and decision-making, etc. The calculation rate and processing efficiency of the existing calculation methods are relatively slow, but they can be solved by the technologies of cloud computing and big data analysis^21^. However, at present, the data environment is complex and redundant, and the structure is diverse. The steps of analyzing and discriminating all kinds of information are tedious, and the real-time response characteristics of information are difficult to meet, which has a certain impact on the stability of energy transmission and the reliability of intelligent control in distribution network^22^. Compared with the feature that cloud computing can be processed remotely and uniformly in the master station, edge computing extends from cloud computing to intelligent terminals and edge nodes of the Internet of Things, and its main purpose is to realize the functions of real-time collection and calculation of localized data information, online diagnosis, ms-level rapid response and precise control of controlled nodes. A single distribution terminal adopts edge calculation, so that data can be calculated and solved on the edge side, and the solution results can be uploaded to the cloud center, which is more conducive to real-time monitoring of the running state and quick and intelligent processing of data. Traditional control is to control the electrical signal, while edge calculation is to control the information. It integrates the key capabilities of communication networks, computing preprocessing, data storage and application programs with edge nodes as the core^23^. At present, all industries are striving to promote the integration of operational, information, communication (OICT) proposed by the Edge Computing Alliance^19^, but in fact, with the wide application of big data and other technologies, the transformation from OICT to data technology (DT) will also be realized, and finally digital automation technology (AT) and intelligent control services will be provided. Figure 1 shows the schematic diagram of data positioning for edge calculation.

### Connecting edge computing with Internet of Things and distribution network

The traditional distribution network can usually be divided into the main station, sub-stations and terminal layers. With the deepening reform of the power network and the massive access of distributed energy, the traditional distribution network has also changed from the passive distribution network with unidirectional transmission to the active distribution network with bidirectional transmission capacity^24,25^, which also makes the distribution system look forward to the change. At present, with the large-scale power electronic equipment and intelligent controllable devices put into use, there are some shortcomings in the traditional control technology and power communication grid. Only by introducing the IoT technology into the power grid can we meet the demand of new distribution system^26^. At present, all layers of distribution equipment in the new distribution network are combined with embedded systems, which makes the traditional distribution network form information interaction with other equipment in the station area based on information communication network, thus constructing a large-scale distribution Internet of Things with physical network, information equipment and computing units coupled. Finally, it can further promote the integration of edge computing, IoT and distribution network, and make the distribution network realize intelligent control at different levels.

## Multi-terminal node distribution model of Internet of Things

**Figure 2:**
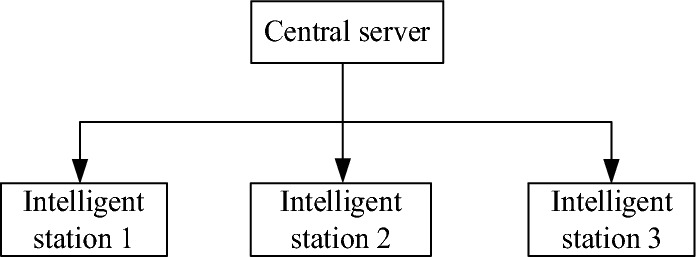
Data and information sharing schematic diagram.

In the distribution network, distribution intelligent terminals can be used as edge computing nodes, with computing, storage and communication functions. The terminal of the smart platform can manage the connected communication devices, sensing devices, measurement devices, and execution devices, and can seamlessly connect to the data center and cloud platform. Through various appropriate communication means, the measurement data is collected, and the local data processing and analysis decisions are carried out. The adjacent intelligent station terminals can form distributed computing nodes, and the measurement data and processing results of the edge nodes of each distributed terminal intelligent device can be shared. Through various suitable communication means, measurement data is collected, and local data processing and analysis are carried out. The terminals in the adjacent intelligent station area can form distributed computing nodes, and the measurement data and processing results of the edge nodes of the intelligent devices of each distributed terminal can be shared. The schematic diagram of device data and information sharing is shown in Fig. 2:

In this diagram, the central server acts as a centralized management and coordination role. Each smart station terminal is connected to a central server and communicates with other terminals.

Data sharing can be achieved in the following ways:

1. Data upload: The intelligent station terminal can upload the collected measurement data and processing results to the central server. These data can include sensor readings, algorithm outputs, etc. A central server is responsible for storing and managing this data, making it accessible to other terminals.
2. Data download: The terminal can download data uploaded by other terminals from the central server. In this way, each terminal can obtain data from the entire distributed network for further processing and analysis.
3. Data sharing: Data can also be shared directly between terminals without going through the central server. This method can improve the data transmission speed and reduce the network load. Terminals can send data to other terminals through point-to-point communication or multicast.
4. Collaborative computing: Distributed terminals can use collaborative computing technology to divide tasks into sub-tasks and execute them in parallel on each terminal. Each terminal is responsible for processing part of the data and passing the results to a central server or other terminal for further integration and analysis.

Through these ways, the adjacent intelligent station terminals can realize the sharing of data and information, thus forming a distributed computing node, providing more powerful computing and analysis capabilities.

**Figure 3:**
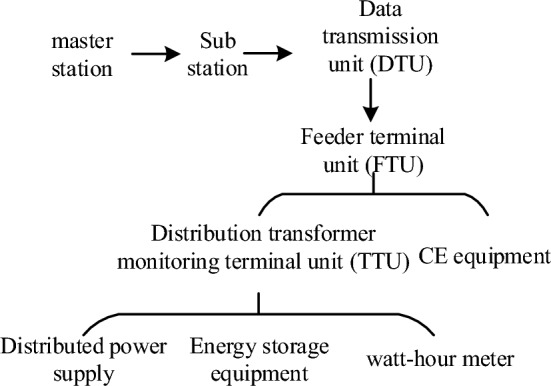
Edge technology structure diagram.

Power distribution IoT is an extension of the IoT technology in the field of power distribution networks. The characteristics of power distribution IoT cannot be reflected through the traditional 3-layer physical architecture (master station, substation, terminal). In order to reflect the characteristics of distribution Internet of Things, this paper designs a distribution Internet of Things architecture based on the PTN network model according to the PE-P-CE structure proposed by the PTN network, combined with the characteristics of edge computing technology to reduce the computation amount and the information interaction of the IoT. Distribution IoT is mainly composed of three types of devices, namely CE devices, PE devices and P devices. The structure diagram of edge technology is shown in Fig. 3.

The CE devices are terminal edge unit level IoT devices located in the active distribution network in the PTN access layer, which can realize self-sensing, self-computing, interchangeable, extensible and self-determining functions. The CE devices in the distribution IoT mainly include distribution transformers, terminal feedback units, intelligent monitoring units, data transmission units, centralized processing units, switch devices, and configured communication modules. The CE devices carry out real-time monitoring, data collection, and intelligent control of terminal devices. Distribution transformer monitoring terminal units monitor the status of distributed power supplies, energy storage devices, and electricity meters. The sub-station device is the edge access device installed in the PTN convergence layer, which plays the role of convergence and access of all edge data. It is called the electronic distribution station in the IoT at the active distribution network system level. It can organize and configure the control instructions of the distribution network by itself, and make self-decisions and optimizations. Multiple CE devices are pooled and connected to PE devices. Through status collection, data interaction, edge analysis and cloud integration, global management in the distribution area is finally realized. As the core layer of the PTN structure, the master station equipment can realize comprehensive information perception in the distribution IoT, integrate and process the data of distributed terminal intelligent devices, conduct in-depth analysis, and then make scientific decisions and issue commands. The master station equipment monitors the status of the distribution network in real time, and the sub-station equipment uploads the information required to be uploaded by distributed terminal intelligent devices to the master station. Moreover, the master station can read real-time data from each CE device, and use the information obtained above to determine the operation status of the smart distribution network.

## Distributed Internet of Things management model based on node collaborative computing

### Construction of collaborative management and control model for Internet of Things

At present, it is difficult to solve the demand of multi-node distribution Internet of Things scenarios by adopting centralized single architecture scheme. Edge computing mode is introduced, and the number of intelligent devices in distribution network terminals is set as edge nodes. Edge computing is the first way to preprocess data. For the edge node management function undertaken by the cloud server, the cloud edge collaboration mechanism can ensure the low delay of data interaction between the distribution network devices. In practical applications, the communication delay of data transmission between devices is still quite high and may even exceed the calculation delay. The following strategies can be used to improve the communication delay of data transmission between devices. First, we can consider optimizing the network topology to ensure more efficient data transmission paths. By carefully designing the network topology, we can reduce the transmission distance of data packets, thus reducing communication delays^27^. Secondly, increasing network bandwidth is another effective measure. Increasing bandwidth can increase data transfer rates and reduce communication delays. This can be achieved by upgrading network hardware or using higher-performance communication equipment^28^. To further optimize communication performance, caching and prefetch technologies can be introduced. By implementing a caching mechanism on the device side, frequent access to the cloud can be reduced, thereby reducing communication latency^29^. Additionally, adopting faster communication protocols is also a way to solve high communication latency issues. Selecting appropriate communication protocols improves data transmission efficiency and accelerates inter-device communications^30^.

**Figure 4:**
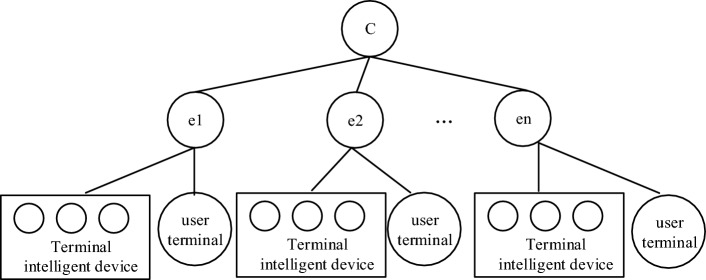
Brief topology of distribution network management structure.

At present, the terminal equipment of the distribution network is complex, including distributed energy, energy storage equipment, flexible load and some controllable equipment. The information interaction of each equipment is mixed, and there is interference in the transmission process. According to the requirements of terminal intelligent devices in the distribution network for request delay and functional integrity, the modeler considers the distribution network and its information system structure, and designs a multi-dimensional collaborative algorithm to meet the requirements. The proposed topology model includes master device node, edge node, user terminal layer and edge device of distribution IoT . The master node C and the edge node set directly managed by the master node C have direct access, and the edge node is provided with its own management center, and the user terminal can access the edge node. A brief topology is shown in Fig. 4.

**Figure 5:**
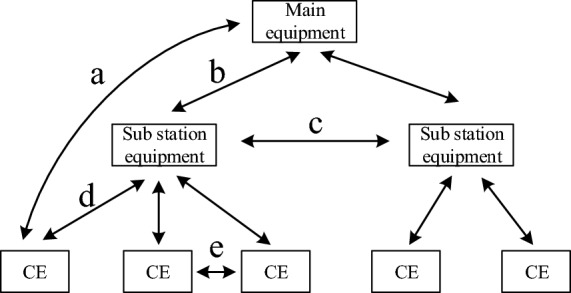
Distribution network management and control structure.

The distribution network is managed hierarchically, and the multi-level centralized and distributed combination of global, intermediate and local control structures is adopted to construct the distribution network management and control structure, as shown in Fig. 5. Based on this structure, first of all, tasks are generated by terminal devices and sent to nodes in the cloud to perform processing computation.

The interface definitions in the Fig. 5 are shown in Table 1.

**Table 1:** Data interface definition.

Data interface	Interface definition
a	Control data is exchanged between CE device and master device
b	Exchange control data between substation equipment and master equipment
c	The sub-station devices exchange data with each other
d	Exchange control data between CE equipment and substation equipment
e	CE exchanges data between devices themselves

According to the physical structure of the distribution network system, the management and control model is used to represent it hierarchically. The various types of equipment and control modes in the model have the following characteristics:

1. Multiple terminal devices can realize information interaction and real-time analysis.
2. In the same structure layer, the effects of different control modes are not coupled and separated from each other, so that the control objectives can be achieved independently, and the influence among their control effects can be ignored.
3. When the superior control fails, each subordinate control will not respond, and it can continue to work according to the operation rules.

### Multi-node collaborative computing method for minimizing effect time target

The traditional load balancing strategy is mainly used in parallel systems. For external data requests, it distributes tasks to multiple processing units, but there are also some shortcomings, such as poor dynamic performance of the algorithm, insufficient consideration of the real-time service capability required by the processing units, and generation of task response and scheduling decisions^31^. Literature^32^ adopts the dynamic equilibrium strategy of linear regression, which can predict the real-time data of terminal edge nodes and then feed back, but it can't meet the coordination needs of cloud and edge because it doesn't consider the propagation delay. Therefore, taking into account the computing capacity of nodes and the propagation delay between edge and cloud centers, a collaborative computing model involving multiple nodes is adopted to minimize response time and achieve collaboration between cloud computing and edge computing. By implementing reasonable task division, node collaborative computing, task scheduling and optimization, as well as error processing and fault tolerance mechanisms, this multi-node collaborative computing model can effectively enhance task execution efficiency and system performance^33–35^.

According to the collaborative architecture model of master device and edge computing described in Section "Multi-terminal node distribution model of Internet of Things", the algorithm is described with the minimum delay as the goal. The data interaction of terminal intelligent devices reaches the edge node first, and the edge node makes the action decision through collaborative control, which involves the round-trip delay between the edge and the master device, the terminal computing processing delay of the master device, and finally the data processing can be completed only after the edge node calculates the delay. Specific parameters to be considered are shown in Table 2.

**Table 2:** Minimum delay parameter description.

Parameter name	Description of parameter
T_edge_	Delay of terminal data transmission to edge nodes
D_edge_	Edge computing time delay
T_cloud_	One-way propagation delay of edge data transmission to master device
D_cloud_	Main equipment calculation time delay

For the data exchange task of terminal equipment, the proposed algorithm is represented by a quaternary data set:

1 $$J = \left\{ {j_{i} |j_{i} = \left( {st_{i} ,c_{i} ,\lambda_{i} ,\mu_{i} } \right)} \right\}$$

where *st*_*i*_ is the number of subtasks included in the *j*_*i*_ task, *c*_*i*_ is the clock cycle required to complete the target task, *λ*_*i*_ is the weight of *j*_*i*_ task in computing resources, and *μ*_*i*_ is the weight of *j*_*i*_ task to computing resources.

The main equipment and edge nodes are characterized by ternary data sets:

2 $$CN = \left\{ {cn_{k} |cn_{k} = \left( {cpu_{k} ,mem_{k} ,t_{k} } \right)} \right\}$$

where *cn*_*k*_ is the corresponding node calculated, *cpu*_*k*_ is the vacancy rate of computing resources, *mem*_*k*_ is the idle rate of computing memory during node calculation, and *t*_*k*_ is the time for a single terminal intelligent device to run independently on the corresponding edge computing node.

Based on the above definition, the cloud computing time can be quantitatively analyzed with the minimum delay as the goal. When the edge node *k* performs the data interaction task *i*, the calculated time delay is:

3 $$D_{edge} (i,k) = (\frac{{\lambda_{i} }}{{cpu_{i} }} + \frac{{\mu_{i} }}{{mem_{i} }}) \cdot st_{i} \cdot \frac{{c_{i} }}{c} \cdot t_{k}$$

When the main device executes the data interaction task *i*, the calculation time delay is:

4 $$D_{cloud} (i,0) = (\frac{{\lambda_{i} }}{{cpu_{0} }} + \frac{{\mu_{i} }}{{mem_{0} }}) \cdot st_{i} \cdot \frac{{c_{i} }}{c} \cdot t_{0}$$

Then, it can be found that the request response time *RT*_*edge*_(*i*,*k*) of the edge node is:

5 $$RT_{edge} (i,k) = 2T_{edge} + D_{edge} (i,k)$$

When the master node executes the data interaction task, the request response time *RT*_*cloud*_(*i*,0) is:

6 $$RT_{cloud} (i,0) = 2(T_{edge} + T_{cloud} ) + D_{cloud} (i,k)$$

In order to minimize the response time of the request, the method adopted is to choose the local edge and the main device node with short response to perform this task. After the task is submitted, the scheduling decision is made by the edge node first. In order to judge which node is the task executor, *T*_edge_ is removed from the calculation response time for comparison during the decision-making. The judgment formula is as follows:

7 $$\left\{ \begin{gathered} 2T_{could} + \left( {\frac{{\lambda_{i} }}{{cpu_{0} }} + \frac{{\mu_{i} }}{{mem_{0} }}} \right) \cdot st_{i} \cdot \frac{{c_{i} }}{c} \cdot t_{0} \hfill \\ \ge \left( {\frac{{\lambda_{i} }}{{cpu_{k} }} + \frac{{\mu_{i} }}{{mem_{k} }}} \right) \cdot st_{i} \cdot \frac{{c_{i} }}{c} \cdot t_{0} , \hfill \\ 2T_{could} + \left( {\frac{{\lambda_{i} }}{{cpu_{0} }} + \frac{{\mu_{i} }}{{mem_{0} }}} \right) \cdot st_{i} \cdot \frac{{c_{i} }}{c} \cdot t_{0} \hfill \\ < \left( {\frac{{\lambda_{i} }}{{cpu_{k} }} + \frac{{\mu_{i} }}{{mem_{k} }}} \right) \cdot st_{i} \cdot \frac{{c_{i} }}{c} \cdot t_{0} , \hfill \\ \end{gathered} \right.$$

**Figure 6:**
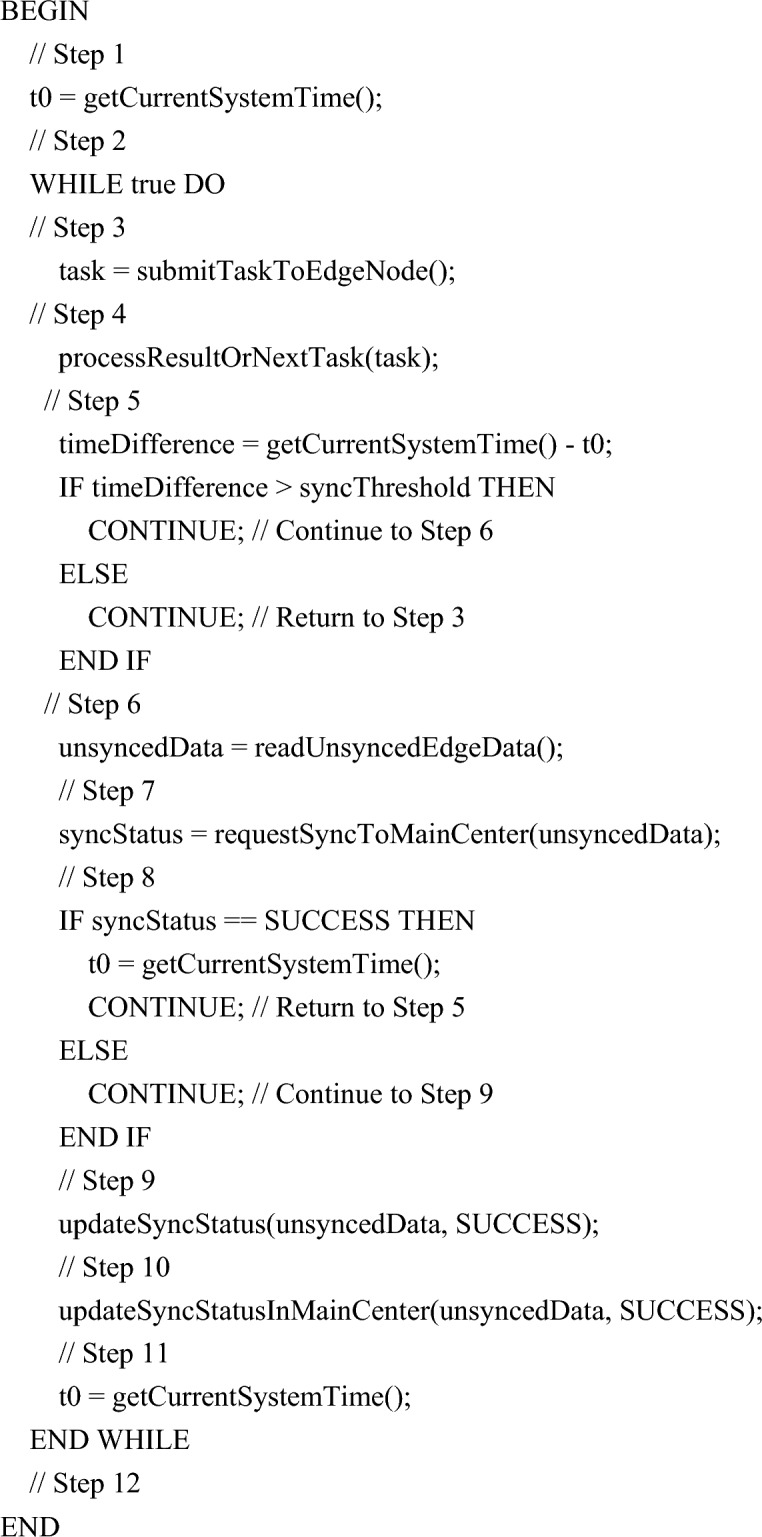
The specific process of data collaboration.

There are two data transmission flows from edge node to master device node and from master device node to edge node in data collaboration, all of which are acted by each edge node's own collaboration controller. The specific process of data collaboration is shown in Fig. 6.

**Figure 7:**
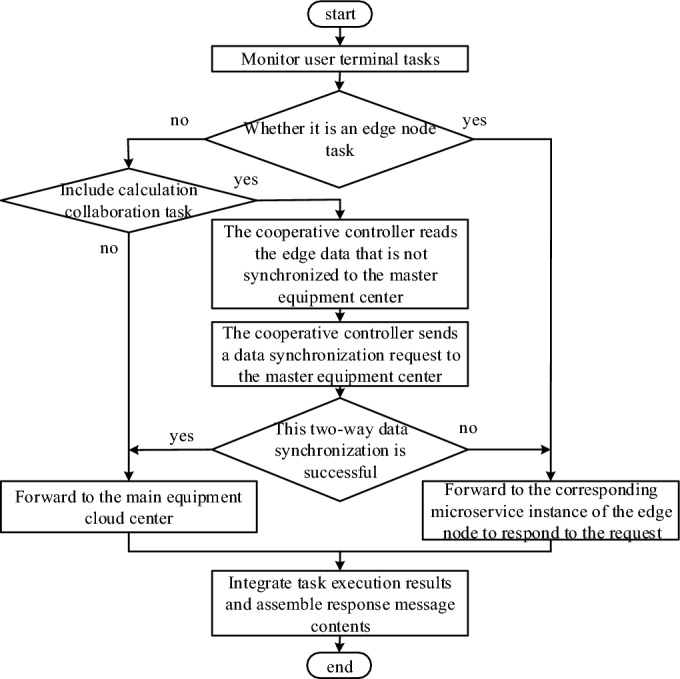
The specific process of service collaboration.

Actually, edge nodes in the system only undertake some application functions. When a user terminal accesses the system, the edge node and the master node need to jointly respond. The master device node is responsible for the global management of the distribution IoT in the whole region, and provides storage capacity for edge nodes. Edge nodes realize device management and control, collaborative decision-making and task execution, and jointly provide application layer services for user terminals.For the tasks submitted by the terminal at the edge node, the collaborative controller of the edge node first adopts scheduling decision or task forwarding according to the task type. The specific workflow is shown in Fig. 7. Based on the above scheme, the distribution network is partitioned, the edge nodes are determined after numbering each distributed terminal device, and the deployment scheme is constructed with the minimum delay as the goal. In the process of reasoning task, the distance between information generating unit and edge nodes, communication environment and other factors will affect the final output result. In order to further reduce the delay, deep neural network (DNN) and equipment edge synergy reasoning algorithm can be introduced. Divide DNN first, reasonably use computing resources to reduce computing complexity and redundancy of edge servers. By training DNN models with different capacities and multiple nodes, select DNN models suitable for application requirements, thus reducing computing burden and total delay. During normal operation, with the different running time, the output data of different DNN layers are also different, which shows great heterogeneity. In practice, the layer module running for a long time may not output data efficiently. Therefore, the DNN is divided into two parts, and the redundant and complicated part can be calculated in the server first in a low transmission efficiency mode, thus reducing the end-to-end waiting time.

## Simulation experiment

Firstly, the proposed collaborative reasoning algorithm is simulated and verified on the cloud simulation platform. In the simulation experiment, a cloud computing center and three edge nodes are set up, and 30 groups of computing tasks are set up. Each group of computing tasks has three task units of the same type, and they are submitted to the three edge nodes respectively, and each group of computing tasks is separated by 1 ms to simulate the change of transmission delay in the actual working process. In the process of simulation, the time delay of service agent module is changed to simulate, and the effect of task coordination processing submitted to edge nodes is simulated and compared.

**Figure 8:**
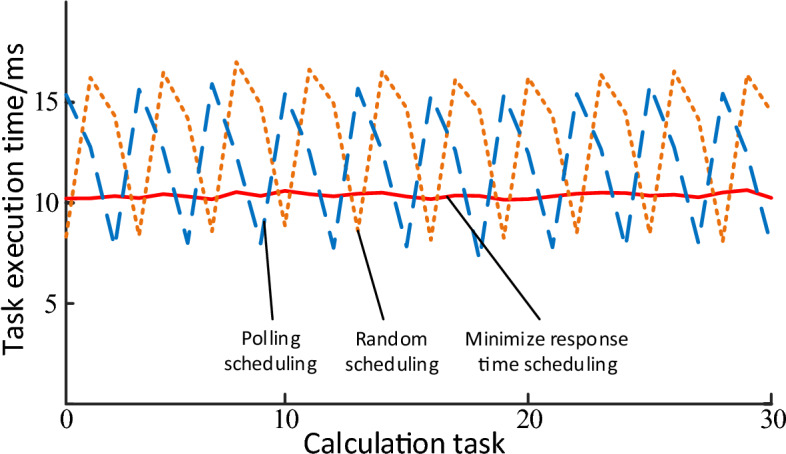
Comparison of results of different scheduing algorithms.

Figure 8 depicts the execution time of different computing tasks submitted to edge nodes under different scheduling methods. The solid line represents the collaborative reasoning algorithm of minimizing response time proposed in this paper, the short dotted line represents the polling scheduling algorithm, and the long dotted line represents the random scheduling algorithm. The results show that the method based on minimizing response time has stable task completion time and relatively short execution time.

**Figure 9:**
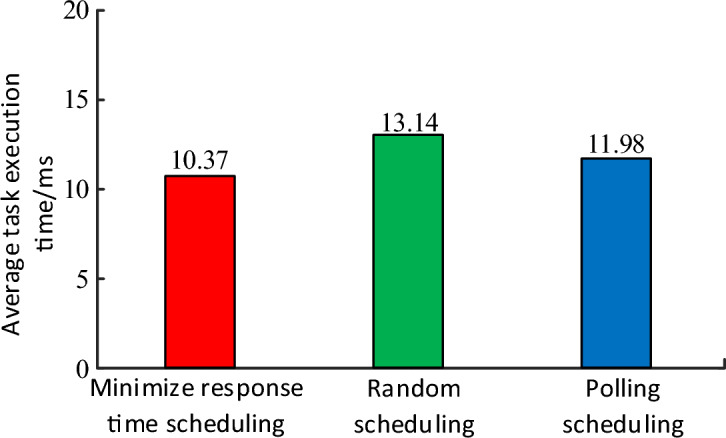
Comparison of the average execution time of different scheduing.

Average the data in Fig. 8, and the average execution time of different methods is shown in Fig. 9. The results show that this method has the shortest average execution time.

As can be seen from Figs. 8 and 9, the system efficiency can be improved by reducing the time for users to submit tasks to edge nodes. This is mainly because the algorithm fully considers the computing capacity gap between cloud computing center and edge nodes and realizes it through optimized scheduling decisions.

In order to build the heterogeneous edge node network environment, four different edge devices are used: JetsonTX1, Jetson TX2, Jetson TK1 and Jetson NaNo. Each time the network topology is generated, it is randomly assigned to the edge nodes. Firstly, the characteristic state indicators of four edge devices are preprocessed by weight matrix. And the smaller the number, the better the running state. The processing results of state indicators are shown in Table 3.

**Table 3:** Pretreated status index.

Equipment	Jetson TX1	Jetson TX2	Jetson TK1	Jetson NaNo
Index data	0.3792	0.1625	0.0887	0.2350

For different computing performance parameters of four types of equipments, the Paleo framework is used to test and run the delay parameters on the edge nodes of each branch, and the running progress is recorded as shown in Table 4.

**Table 4:** The experiment results.

Model	Equipment			
	TX1	TX2	TK1	NaNo
Before optimization	15.58 ms	8.54 ms	6.14 ms	12.68 ms
After optimization	12.04 ms	7.62 ms	5.98 ms	11.25 ms

Compared with the delay time of existing cloud servers, it can be found that the efficiency has been improved in the later stage of optimization. In the method based on edge computing, the average delay of different networks is simulated, and the bandwidth between nodes is set to 1Mbps. The results are shown in Table 5.

**Table 5:** Comparison of experimental results.

Derivation accuracy	0.6	0.64	0.68	0.72	0.76	0.80	0.84
Derivation delay/ms^2^	67.89	78.87	88.76	99.88	109.33	125.36	130.43
Derivation delay/ms^3^	60.48	70.56	82.11	94.32	100.88	118.78	126.89
Derivation delay/ms^4^	55.11	66.71	76.898	87.45	92.67	109.67	123.23
Derivation delay/ms (This study)	49.98	61.94	70.92	81.11	85.32	102.11	120.06

The test results show that compared with other experiments, when the instructions uploaded by the information generation unit are cancelled from the same partition in the network, the simulation experiment data analysis takes the lowest time with the same inference accuracy.

## Conclusion

Aiming at the problems of heavy computing tasks, excessive reasoning delay, and high redundancy of complex data acquisition, this paper adopts cloud-edge coordination based on edge computing technology and the combination of IoT and intelligent distribution network, and develops coordination strategies through three aspects: computing, data and service coordination. Based on the PTN network structure, a multi-terminal node distribution model of distribution IoT is proposed, and a distributed management model of distribution network IoT based on edge multi-node collaborative computing is established. Finally, the distribution autonomy and collaborative management and control of the distribution network are realized, effectively avoiding the problem of traditional centralized management.

## Data Availability

The research project involved in this study has not yet been completed, therefore, the data is not suitable for publication at this time. If readers need information, please consult with the corresponding author to obtain it.
